# Intracerebral Hemorrhage in a Patient With Newly Diagnosed Immune Thrombocytopenic Purpura Precipitated by a Viral Illness

**DOI:** 10.7759/cureus.57284

**Published:** 2024-03-30

**Authors:** Ann Pongsakul, Amy Daniel, Roddy Lochala, David E Martin

**Affiliations:** 1 Family Medicine, Unity Health, Searcy, USA; 2 Graduate Medical Education, Unity Health, Searcy, USA

**Keywords:** immune thrombocytopenic purpura (itp), internal medicine (general medicine), hematology-oncology, inpatient care, brain bleed, acute hemorrhagic stroke, viral itp, stroke, acute cva, immune thrombocytopenia (itp)

## Abstract

Intracerebral hemorrhage (ICH) is a rare and severe complication of immune thrombocytopenic purpura (ITP) that can be spontaneous. Viral illnesses, other infections, autoimmune disorders, and medications can cause ITP. ITP causes a significant decrease in platelet levels, increasing bleeding risk. ITP can be treated by steroids, intravenous immunoglobulin, plasmapheresis, platelet transfusion, biological agents, and splenectomy. ICH treatment involves the treatment of underlying ITP, as well as any neuro-interventional procedures needed. In this case report, we look at the presenting symptoms and treatment course of an interesting case of ICH in a patient who developed ITP after a viral upper respiratory infection.

## Introduction

Immune thrombocytopenic purpura, or idiopathic thrombocytopenic purpura (ITP), is an autoimmune disorder caused by autoantibodies that attack platelets and megakaryocytes, leading to low platelet levels [1-3]. ITP can be caused by many factors, including infections, autoimmune disorders, and medications [4,5]. Clinically, ITP is characterized by exceptionally low platelet counts (thrombocytopenia) [2]. Most cases are asymptomatic [2]. However, deficient platelets can cause bleeding from gums, bruising, purpuric spots on the body, and, in extremely rare cases, intracranial hemorrhage (ICH) [2]. The incidence of ICH in ITP patients is 1.54% in all ages [6]. ICH occurs in about 0.1% to 1% of children and even less in adults [6,7]. Treatment of ICH in the setting of ITP is challenging and usually involves treatment of underlying ITP and possible neurosurgical intervention. Treatment options for ITP include steroids, intravenous immunoglobulin (IVIG), plasmapheresis, platelet transfusion, splenectomy, and biological agents such as rituximab, azathioprine, and mycophenolate mofetil [2,8-10]. We report a unique case of a previously healthy patient who experienced ICH due to ITP preceded by an upper respiratory infection. The patient developed significant neurological deficits very rapidly within a few hours. Despite the high risk of mortality and barriers leading to delays in diagnosis and treatment, the patient continues to make progress in their recovery. 

## Case presentation

**Figure 1:**
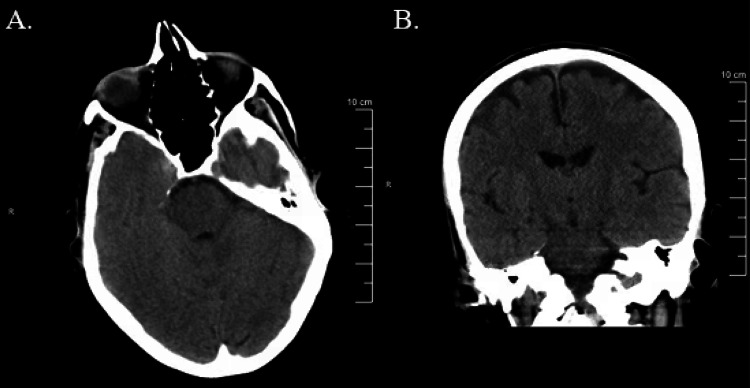
Negative non-contrast CT head axial and coronal view, respectively, at the time of initial presentation in the ED. No focal neurological deficits were present at that time.

A 51-year-old female presented to the emergency department (ED) with vague complaints of abdominal pain, nausea, and vomiting, worsening confusion, as well as complaints of left-sided numbness in her face and upper and lower extremities. Medical history was only significant for iron deficiency anemia on iron supplementation. The patient reported having flu-like symptoms for seven days before coming to the ED. She denied any trauma. Initial vital signs were stable. Physical exams performed by the emergency physician in the afternoon and the admitting physician at approximately 19:00 were unremarkable for neurological deficits. The patient did not have any signs of purpura. Initial labs were remarkable for a hemoglobin level of 6.7 g/dL and platelet level of 29 th/ul. Haptoglobin was 170 mg/dL, lactate dehydrogenase was 358 U/L, PT/PTT was 11.3/24.1 seconds, respectively, and fibrinogen was 145 mg/dL, all within normal limits. Urinalysis was remarkable for 4+ bacteria. A non-contrast CT of the head (NCCT) showed no acute intracranial abnormality (Figure 1). The patient was admitted to the general floor for thrombocytopenia and encephalopathy. The patient was given one unit of packed red blood cells as a treatment for anemia, and Rocephin was started for a urinary tract infection.

**Figure 2:**
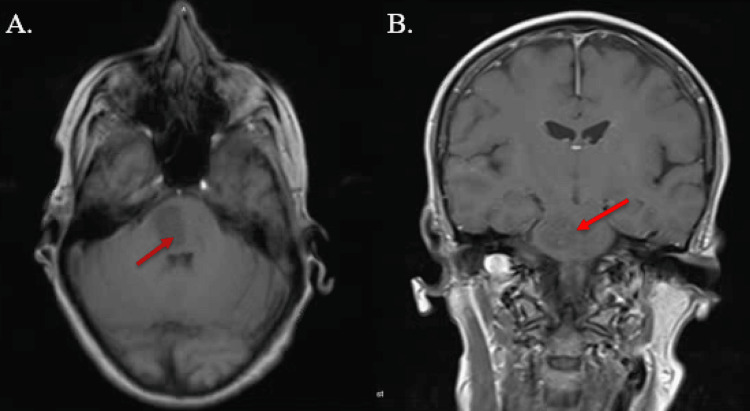
MRI axial and coronal views, respectively, showing intraparenchymal hemorrhage at the level of the pons measuring 2.7 x 1.8 cm with significant edema at about 18 hours after presentation in the ED. The patient had signs of significant left-sided hemiparesis, left-sided facial droop, fixed leftward gaze, and slurred speech at that time.

**Figure 3:**
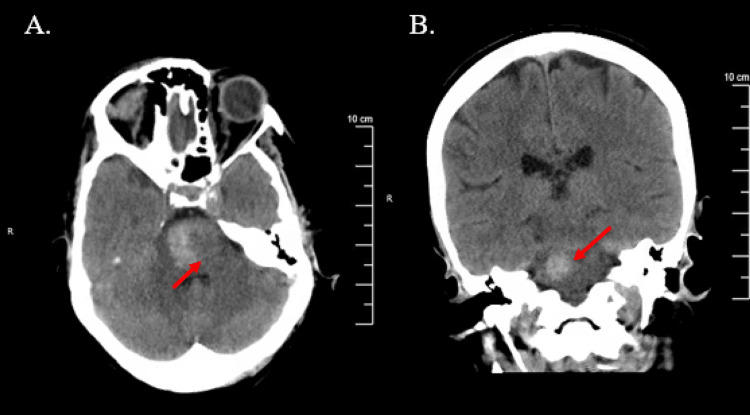
Non-contrast CT head axial and coronal views, respectively, showing the area of intracranial hemorrhage measuring 2.5 x 1.2 cm to the right side of the pons 24 hours after presentation in the ED. The patient still had significant neurological deficits at that time, but no change in severity.

By 21:00 that evening, the patient had developed significant neurological deficits that were only seen by nursing staff, who were under the impression that these had been present since admission. On exam the next morning, the patient was found to have significant left-sided paralysis in the upper extremity and lower extremities, left-sided facial droop, fixed leftward gaze, slurred speech, and inability to void urine. CT angiogram (CTA) of the head and neck shortly after showed no evidence of occlusion or aneurysm. Morning labs showed platelet levels decreased to 17 th/ul, though hemoglobin levels responded appropriately to red blood cell transfusion at 10.9 g/dL. A peripheral smear was done, which showed no schistocytes. ITP was highly suspected due to a recent viral illness. Iron studies, B12, folate, and repeat lactate dehydrogenase were unremarkable. Brain MRI was performed with contrast at about 18 hours after presentation in the ED (Figure 2) and a second NCCT was performed at about 24 hours after presentation in the ED (Figure 3), showing evidence of intraparenchymal hemorrhage measuring 2.7 x 1.8 cm involving the brain stem with edema of the pons.

After consultation with neurology and neurosurgery at nearby tertiary medical centers, tranexamic acid, dexamethasone, and platelet transfusion were started. Repeat platelet levels after transfusion were 60 th/ul. There were no indications for immediate transfer for a neurosurgical procedure at that time due to the risk of increased intracranial bleeding. Multiple attempts were made to transfer the patient to several facilities with available plasmapheresis, inpatient neurology, and neurosurgery, but these were unsuccessful.

Despite being hemodynamically stable since admission, there was a concern for the patient to decompensate with rapidly evolving neurological deficits. She was transferred to the critical care unit for closer monitoring and then to the nearest tertiary medical center. After receiving one week of IVIG and glucocorticoids for ITP, the patient was transferred back to our facility with stable platelet levels at 301 th/ul. Due to the inability to swallow, the patient was started on nasogastric tube feeds at the outside facility and then received percutaneous endoscopic gastrostomy (PEG) tube placement at our facility for longer-term nutrition. The patient was discharged to an inpatient rehabilitation facility, and after a few weeks, she was discharged home. The patient is now tolerating foods by mouth with some improvement in speech and left-sided paralysis. 

## Discussion

The development of ITP is rare in itself [7,11]. The median age for adult ITP patients is 50-55 years, and middle-aged females are more likely to develop ITP than males; however, after age 60, males are more likely to develop ITP [11]. The development of spontaneous ICH in ITP is infrequent [7]. One retrospective case-controlled study found an ICH incidence of 1.54% in all ages with ITP [6]. ITP can be classified into three groups based on chronicity: 1) newly diagnosed, up to three months; 2) persistent, between three and twelve months after diagnosis; and 3) chronic, twelve months after diagnosis [5,11]. The spontaneous remission of bleeding symptoms is less common in the persistent and chronic phases [5,10,11]. ITP clinical presentations vary greatly. Patients may experience initial symptoms of bleeding gums, nosebleeds, and petechiae throughout the body while some ICH cases occur without any of these initial symptoms, as with our patient whose first symptoms of bleeding were her neurological deficits [4,7]. Some cases are gradual and become chronic, and some are abrupt and acute in onset [2]. In a meta-analysis of seven ITP patients who developed subdural hemorrhages, the mean length of time since diagnosis was 2.3 months to 8.3 years [1].

It was unknown whether our patient’s upper respiratory symptoms that occurred several days before presentation were due to COVID-19 or influenza. However, the patient tested negative for both on admission. The pathogenesis of ITP involves dysregulation of the immune system. This can stem from many factors, such as viral infections, rheumatological disorders, leukemia, and immune deficiencies, which lead to antibodies directed against the platelet glycoprotein IIb/IIIa that impair platelet formation and destroy platelets [3,4,12]. Viral infections associated with ITP include HIV, hepatitis C, rubella, mumps, varicella, Epstein-Barr virus, and COVID-19. Thrombocytopenia has been linked as a complication of COVID-19 [12]. The overactive immune system can also affect platelet formation in the bone marrow and cause platelet aggregation from lung injury [12,13]. In a recent meta-analysis, the severity of COVID-19 and thrombocytopenia were found to be directly proportional to each other [12]. In two case reports reviewed, two patients were found to have ITP after severe COVID-19 infections [5,14]. In one such case in France, the patient also developed a subarachnoid microhemorrhage in the right frontal lobe without significant neurological deficits and recovered well after receiving prednisolone and eltrombopag [14].

The gold standard for diagnosing hemorrhagic stroke is NCCT [15]. One case report reviewed involved a female patient who presented for ischemic stroke, and NCCT did not show cerebral bleeding; however, a microbleed was found on susceptibility-weighted imaging MRI [15]. The patient was considered for thrombolytic therapy initially, but it was not administered [15]. Another reported case involved a hypertensive thalamic hemorrhage not initially detected by NCCT [15]. However, it was found on MRI 12 hours later, and the third case found cerebral hemorrhage on MRI 3 hours after the initial NCCT was negative [15,16]. In our case, the patient’s only initial neurological symptoms were numbness to the left upper and lower extremities, which were not concerning due to negative NCCT. However, severe neurological deficits developed unnoticed through the night and, upon examination in the morning about 12 hours later, prompted a brain MRI. In addition, the occurrence of hemorrhagic stroke not seen on NCCT without contrast raises the need for consideration of alternative imaging options for diagnosis, and relying on NCCT-based thrombolytic treatment may not be safe [15]. 

Based on the analysis of our case and several outside cases, we can conclude the importance of increasing awareness of ICH as a complication of ITP and the importance of management and prompt management of the ICH and ITP itself [1,4,5,7,9,12-16]. In severe cases as in our patient, a neurosurgical intervention was not indicated due to the risk of further bleeding, therefore the focus was on rapidly treating the ITP and raising platelet levels to prevent further bleeding. Currently, expertise in the management of severe bleeding in ITP is limited, as most cases of ITP are asymptomatic [11] as in our patient who did not show signs of bleeding initially. The mortality rate of these cases varies from 25%-55% [5,17]. Management is stepwise, based on the severity of the bleeding, and focused on treating the underlying cause, preventing the immune system from attacking platelets further, and increasing platelet levels. Typically, the first line is steroids, but the response time can be two to six days [2,8]. Therefore, alternatives such as IVIG, plasmapheresis, biological agents, and splenectomy may need to be considered to prevent further platelet destruction rapidly [5,8,17].

## Conclusions

ICH is a rare but severe complication of ITP that is not widely recognized but can occur in patients who have simple viral upper respiratory illnesses or chronic infections. Most people who do develop ITP are symptomatic; however, they have the potential to develop debilitating neurological deficits from ICH. While NCCT is the gold standard for diagnosing ICH, awareness of limitations to detect smaller hemorrhages and considering alternative imaging is essential. Prompt neurosurgical intervention may be required in certain cases of ICH due to ITP; however, prompt ITP treatment and stabilizing platelet levels are also essential for successful recovery.
